# Study on the Characterization of Physical, Mechanical, and Creep Properties of Masson Pine and Chinese Fir Wood Flour-Reinforced High-Density Polyethylene Composites

**DOI:** 10.3390/polym16243507

**Published:** 2024-12-17

**Authors:** Hailong Xu, Xueshan Hua, Yan Cao, Lifen Li, Baoyu Liu, Xiaohui Yang, Hua Gao

**Affiliations:** 1 Special and Key Laboratory for Development and Utilization of Guizhou Superior Bio-Based Materials, Guizhou Minzu University, Guiyang 550025, China; xu00hailong@163.com (H.X.); 18385498826@163.com (X.H.); liubaoyu1020@163.com (B.L.); xiaohuiyang90@163.com (X.Y.); gaohua202406@163.com (H.G.); 2 Engineering Research Center of Green and Low-Carbon Technology for Plastic Application, Guizhou Minzu University, Guiyang 550025, China; 3 College of Forestry, Guizhou University, Guiyang 550025, China

**Keywords:** wood flour-reinforced polymer composite, mechanical properties, creep performance, mathematical model

## Abstract

Improving the physical, mechanical, and creep properties of wood fiber-reinforced polymer composites is crucial for broadening their application prospect. In this research, seven types of high-density polyethylene (HDPE) composites reinforced with different mass ratios of Masson pine (*Pinus massoniana* Lamb.) and Chinese fir [*Cunninghamia lanceolata* (Lamb.) Hook.] were prepared by a two-step extrusion molding method. The mass ratios of the two fibers were 60:0, 50:10, 40:20, 30:30, 20:40, 10:50, and 0:60, respectively. The surface color, density, dimension stability, bending, tensile, impact properties, dynamic mechanical properties, and 24 h creep properties at a 10% stress level of the seven composites were investigated. Additionally, the Rule of Mixtures (ROM), the Inverse Rule of Mixtures (IROM), the Hirsch models, and the improved model were employed to simulate the mechanical properties, while the Findley index model, the two-parameter index model, and the modified ExpAssoc model were employed to simulate the creep performance of the composites. This study revealed that as the proportion of Chinese fir wood flour increased, the mechanical properties of the composites gradually improved, the storage modulus showed an increasing trend, while the loss modulus decreased, and the overall creep strain of the composites increased. Among the various models, the modified model simulated the mechanical properties of the composites the best, while the modified ExpAssoc model simulated the creep behavior most effectively.

## 1. Introduction

Wood–plastic composites (WPCs) are made from wood fiber and polymer. The raw materials for WPCs are widely available and free from the common defects of natural wood, such as cracking and warping. WPCs effectively retain the excellent characteristics of plastic, such as corrosion resistance, waterproofing, and termite resistance [1], while also preserving the appearance, texture, and ease of processing characteristic of natural wood materials. Due to their simple preparation process and outstanding comprehensive performance, WPCs are commonly used in indoor and outdoor flooring, fences, furniture and decorative materials [2,3,4], etc. Additionally, WPCs are recyclable, contributing to significant economic benefits [5]. With increasing environmental awareness and advancements in WPC manufacturing and testing technologies, the applications of WPCs are becoming increasingly widespread [6].

Previous studies have shown that the type of wood flour can affect the physical [7,8], mechanical [7,8,9], and aging resistance [10] properties of WPCs. Masson pine (*Pinus massoniana* Lamb.) and Chinese fir [*Cunninghamia lanceolata* (Lamb.) Hook.], which are wildly distributed in subtropical areas of China [11], exhibit excellent mechanical properties, such as bending, compressive, and tensile strength, making them widely processed and utilized. Utilizing the residual branches and sawdust from the processing of Masson pine wood and Chinese fir wood to reinforce common waste plastics in WPCs cannot only alleviate the increasing scarcity of fossil energy and aligns with the requirements of energy conservation and environmental protection advocated in the new era, but also supports the new growth requirements of the circular economy and green ecological industry in Southwest China, contributing to the achievement of the “dual carbon” goals.

Huang et al. [12] analyzed the mechanical properties of single fibers from Masson pine and Chinese fir. They found that the breaking load, tensile strength, and percentage of breaking elongation of Masson pine single fibers were 315.83 mN, 885.97 MPa, and 3.98%, respectively, whereas those of Chinese fir single fibers were 207.16 mN, 822.20 MPa, and 3.70%, respectively. The study indicated that Masson pine single fibers possess high strength and better ductility compared to Chinese fir single fibers. Wang Z. [13] reported that the total surface free energy and non-polar surface free energy of Masson pine were 27.3 mJ/m^2^ and 35.5 mJ/m^2^, respectively, which were much lower than those of Chinese fir (42.4 mJ/m^2^ and 41.6 mJ/m^2^). Additionally, Masson pine wood had a much higher surface polarity (11.0 mJ/m^2^) compared to Chinese fir, (0.74 mJ/m^2^). Therefore, the interfacial bonding strength between Masson pine wood and plastic in WPCs was relatively weak, and the water resistance at the interface was poor. Furthermore, the small molecule resins present in the cells of Masson pine wood tend to form a boundary layer on the surface, further reducing the internal bonding strength of the WPCs. Cao et al. [14] studied the physical and mechanical properties of WPCs made from Masson pine and Chinese fir using a two-step extrusion method. The results showed that the water absorption thickness expansion rate of composites made from Masson pine fibers was 3.43 times greater than those of composites made from Chinese fir fibers, whereas the mechanical properties of the composites made from Chinese fir fibers were significantly superior to those made from Masson pine fibers. Zhao et al. [15] investigated the physical, mechanical properties, and anti-mold properties of polyethylene composites reinforced with seven types of plant fibers, including Masson pine wood and Chinese fir fibers. The study found that the water absorption rate and thickness expansion rate of composites made from Masson pine fibers were higher than those of composites made from Chinese fir fibers. While the bending and impact mechanical properties of the composites from both fiber types were similar, the anti-mold performance of polyethylene composites reinforced with Chinese fir fiber was much better than that of Masson pine fiber. These findings suggest that, compared to Masson pine, Chinese fir is a more promising reinforcement material for WPCs.

The microstructure of WPCs is complex and changeable, so it is necessary to use simple and accurate mathematical models to explain the influence of the structure of the composites on the related properties. The commonly used models for predicting properties of the composites are the Rule of Mixtures (ROM) [16,17], the Inverse Rule of Mixtures (IROM) [18], and the Hirsch model [19,20]. In addition, researchers have used other models to describe various composite properties based on different parameters, such as the Bowyer–Bader model [20], Composite Cylinder Assemblage model [21], Halpin–Tsai model [17], Hashin–Rosen model [22], Kelly–Tyson model [23], Levin model [24], Mori–Tanaka model [25], the Proportional Hazards model [26], Cox Shear Lag model [16], and the two-parameter Weibull Distribution model [27].

Cao et al. [26] evaluated the bending, tensile, and impact properties of HDPE composites reinforced with flax and wood mixed fibers. They applied the ROM, IROM, and Hirsch models to fit the mechanical properties of the composites. Among these models, the IROM model was found to be the most accurate in describing the mechanical properties. The ROM model is based on the assumption of Voigt, which posits that the strain generated by the matrix and the fiber is equal, while the IROM model relies on the assumption of Reuss, which suggests that the stress is equal in both the matrix and the fiber [27,28,29,30].

As a new type of environmentally friendly material, WPCs have a good prospect in the field of biomass composites. However, creep behavior often leads to mechanical failure or even fracture in WPCs, which limits their further development and application. Therefore, research on the creep behavior of WPCs and the prediction of their creep characteristics is of great importance [31,32]. Previous studies on creep models have shown that selecting and constructing appropriate mathematical models is key to better simulating the creep behavior of these materials.

Liu et al. [33] used the four-parameter Burgers model to derive expressions for the effective relaxation modulus and effective Poisson’s ratio of the viscoelastic matrix material in the time domain. Zhao et al. [34] fitted the creep curve of WPCs made from poplar wood flour and found that the fractional order model provided the best fit for creep curves at high stress levels. Dong [35] simulated and analyzed the creep curves of polypropylene WPCs at five different stress levels using a four-element viscoelastic model connected in series by the Maxwell model and Kelvin model. The results showed that the correlation coefficient *R*^2^ between the fitting curve of the four-element viscoelastic model and the creep test curve of polypropylene WPCs was more than 0.98, demonstrating that the four-element model accurately simulates the short-term creep behavior of WPCs. In an effort to predict WPC creep behavior, Abdollah et al. [36] used the Findley index model to fit and analyze the creep curve of WPCs, and proved that the creep behavior of WPCs conforms to the Findley index law. Du et al. [37] used both the Findley index model and a generalized 10-parameter Kelvin model to simulate the 24 h creep curve of WPCs, and the results showed that the generalized 10-parameter viscoelastic model provided a good fit. Zhang et al. [38] used the ExpAssoc function to fit the creep curve of loess through the constitutive five-element Kelvin model, showing that this model effectively describes the creep characteristics of loess.

However, there is limited literature on the properties of different hybrid fiber-reinforced WPCs, and models are rarely used to describe hybrid fiber-reinforced thermoplastic composites.

To fully utilize the wood processing residues from Masson pine and Chinese fir wood in Southwest China, this study used a mixed wood flour from Masson pine and Chinese fir with different mass ratios as the reinforcing phase, and combined it with high-density polyethylene (HDPE) to fabricate WPCs. The effects of the wood flour mass ratio from Masson pine and Chinese fir on the physical, mechanical, dynamic mechanical, and creep properties of the composites were studied. The ROM, IROM, and Hirsch models and the improved model were used to simulate the mechanical properties of the WPCs, while the Findley index model, the two-parameter index model, and the modified ExpAssoc model were applied to analyze the creep properties. Finally, the model exhibiting the best simulation performance for each property was then selected. This study provides a theoretical basis and reference for the safety and application of polymer composites reinforced with dominant wood fibers in Southwest China under load conditions.

## 2. Materials and Methods

### 2.1. Materials

HDPE particles (5000S) as the matrix in composites were purchased from Daqing Petrochemical Company in China. These particles have a melt flow index of 0.35 g/10 min and a solid density of 954 kg/m^3^. Masson pine and Chinese fir wood processing residues (wood flour or powders) with a particle size of 0.18 to 0.85 mm were supplied by a wood processing plant, Guiyang, Guizhou province, China. Maleated grafted polyethylene (MAPE) particles (CMG 9804) were used as a compatibilizer, and wax was used as a processing lubricant. Both of these reagents were purchased from Tianjin Bochen Limited Company in China.

### 2.2. Preparation of the Composites

**Figure 1:**
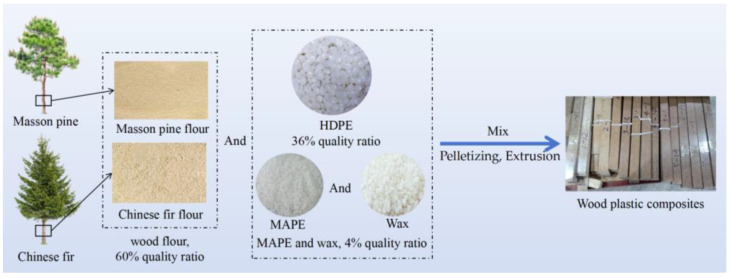
Production flowchart of WPCs.

Prior to mixing with the polymer matrix, the wood flour was dried in an oven (DHG-9140, Shanghai Yiheng Experiment Instrument Co., Shanghai, China) at 105 °C until its moisture content dropped below 3.0%. Dried HDPE, wood flour, and other additives were then mixed in a high-speed mixer (9FQ-300, Dandong Main Fire Machinery Factory, Dandong, China) at 75 °C for 15 min. The resulting mixture was then compounded through a co-rotating twin-screw extruder (SJSH30, Nanjing Rubber and Plastic Machine Ltd., Nanjing, China). The compounded blend was subsequently fed into an SJ 45 mm conical counterrotating single-screw extruder to produce composite lumber samples. The screw speed was maintained at a constant 100 rpm, and the temperature was set between 150 °C and 175 °C during lumber extrusion. After cooling, the compounded material was broken into granules, which were then directly fed into a single-screw extruder (SJ45, Nanjing Rubber and Plastic Machine Ltd., Nanjing, China) to create the lumber, which had a cross-section of 40 mm × 4 mm. A brief production flowchart of WPCs is shown in Figure 1.

Seven different types of WPCs were prepared, with varying mass fractions of raw materials as shown in Table 1. As such, WPC1 was reinforced with Masson pine wood flour, WPC7 with Chinese fir wood flour, while WPC4 was produced with an equal mass ratio of Masson pine and Chinese fir wood flour.

### 2.3. Performance Testing and Characterization

#### 2.3.1. Color Test

A colorimeter (CM-2003d, Konica Minolta, Japan) was employed to measure the brightness (L*), red–green axis chromaticity index (a*), and yellow–blue axis chromaticity index (b*) of the WPCs’ surfaces according to the CIELAB color system. Each sample was measured at five different points, and the average values were recorded. The color differences (∆L*, ∆a*, ∆b*) were obtained by comparing the WPCs’ results to those of the control sample (WPC 1). The total color change (∆E*) was calculated using Formula (1).

(1) $$∆E*=\sqrt{{∆L*}^{2}+{∆a*}^{2}+{∆b*}^{2}}$$

#### 2.3.2. Density Test

The density of the WPCs was tested according to the GB/T 17657-2013 [39] standard. An average value was recorded after testing five samples from each group. The electronic balance model used in the test was LD31001, manufactured by Shenyang Longteng Electronic Weighing Instrument Co., Ltd., Shenyang, China.

#### 2.3.3. Dimensional Stability Test

The 24 h water absorption rate and thickness swelling rate of WPCs were tested according to GB/T 17657-2013 [39]. Samples measuring 20 mm × 20 mm × 4 mm were submerged in a water bath at 20 °C for 24 h. Afterward, their surfaces were carefully wiped with a cloth, and measurements of mass and thickness were recorded. Each group consisted of 5 specimens, and the average values were recorded.

The 24 h water absorption rate (*W*) of the sample was calculated by the following formula:

(2) $$Water\;absorption/\%=\frac{W_{t}-W_{0}}{W_{0}}\times 100\%$$

In Formula (2), *W*_0_ and *W_t_* are the weights of the WPCs before and after immersion, respectively.

The 24 h water absorption thickness swelling rate (*T*) of the sample was calculated by the following formula:

(3) $$Thickness\;swelling/\%=\frac{T_{t}-T_{0}}{T_{0}}\times 100\%$$

In Formula (3), *T*_0_ and *T*_t_ are the thicknesses of the WPCs before and after soaking, respectively.

#### 2.3.4. Mechanical Properties

(1) Bending Performance

Three-point bending tests were performed using a universal electronic testing machine (RGT-20A, Shenzhen Ruel Instrument Co., Ltd., Shenzhen, China) to evaluate the bending strength and modulus of WPCs. Samples measuring 80 × 13 × 4 mm were used for these evaluations. The tests were conducted with a span of 64 mm and a loading speed of 1.9 mm/min, following ASTM D 790-2003 standards [40]. Five samples were set for each group, and the results were averaged.

(2) Tensile Performance

The tensile properties of the composite samples were conducted according to ASTM D 638-2010 [41] using a universal electronic testing machine (RGT-20A, Shenzhen Ruel Instrument Co., Ltd., Shenzhen, China) with a span of 50 mm and a tensile speed of 5 mm/min. The specimen is dumbbell shaped, 165 mm × 20 mm × 4 mm (the narrowest part is 12.7 mm wide). Five specimens were tested in each group and averaged.

(3) Impact Performance

The simple supported beam pendulum impact tests of the composite samples were conducted on the beam impact testing machine (XJ-50G, Hebei Chengde Mechanical Experimental Machine Co., Ltd., Chengde, China) according to the guidelines in GB/T 1043.1-2008 [42]. The specimens had a length of 80 mm, a width of 10 mm, and a thickness of 4 mm. The test span was set as 60 mm, with an impact speed of 2.9 m/s, and a pendulum energy of 2 J. Five specimens were tested in each group and averaged.

#### 2.3.5. Dynamic Thermomechanical Analysis

The dynamic thermodynamic properties of WPC samples were measured using a three-point bending mode. The tests were conducted over a temperature range of 20–120 °C, with a heating rate of 5 °C·min^−1^, at a frequency of 1 Hz. The sample size was 40 mm × 10 mm × 3 mm. Two samples were tested for each group of composites.

#### 2.3.6. Creep Performance

(1) Bending limit load test

In order to study the effect of the wood flour content of Masson pine and Chinese fir on the bending creep properties of WPCs, the bending limit loads of seven kinds of WPCs were first determined at normal temperature. The specimens measured had a length of 100 mm, a width of 40 mm, and a thickness of 4 mm. The test span was 64 mm, and the speed was set to 1.9 mm/min. The test was repeated five times for each WPC sample, and the average value was calculated as the test result.

(2) Bending creep performance test

The bending creep properties of seven kinds of WPCs were tested according to ASTM D 2990-2001 [43]. The tests were conducted at room temperature, with humidity maintained between 55% and 65%. A three-point bending loading method was adopted, with a span of 64 mm. A constant vertical downward force equivalent to 10% of the limit load was applied to the specimens. The deformation of the samples at different times was measured using a dial gauge. The specimens were rectangular and had the same size as those used in the bending limit load tests. Each group of tests was repeated three times, and the measured size was used in the calculations. The shape variable (*D*t) of the material at time *t* was determined by calculating the difference between the micrometer reading before loading (time *t*_0_) and at time *t*. The relationship between stress and load is shown in Equation (4).

(4) $$\sigma =\frac{3PL}{2bd^{2}}$$

The relationship between deformation and strain is shown in Equation (5).

(5) $$\varepsilon =\frac{6Dd}{L^{2}}$$

In the above equations, *P* is the applied load (N), *D* is the deformation (mm), *L* is the span (mm), *σ* is the stress (MPa), *b* is the width of the specimen (mm), *ε* is the strain (mm/mm), and *d* is the thickness of the specimen (mm).

#### 2.3.7. Simulation and Comparison of Mechanical Properties

(1) Simulation of Mechanical Properties

The ROM model, IROM model, and Hirsch model were applied to predict the mechanical properties of the WPCs.

The formula for the ROM model is as follows [28,44]:

(6) $$P_{c}=\alpha P_{1}V_{1}+P_{2}V_{2}$$

The formula of the IROM model is as follows [27,44]:

(7) $$P_{c}=\frac{P_{1}P_{2}}{P_{1}V_{2}+P_{2}V_{1}}$$

The Hirsch model is produced by combining the ROM and IROM models. The formula of the Hirsch model is as follows [27,44,45]:

(8) $$P_{c}=\beta \left(P_{1}V_{1}+P_{2}V_{2}\right)+\left(1-\beta \right)\frac{P_{1}P_{2}}{P_{1}V_{2}+P_{2}V_{1}}$$

In the formulae above, *P* stands for mechanical property value, and *V* represents the volume fraction (%). The subscript 1 represents the Masson pine wood flour-reinforced HDPE composite, while the subscript 2 indicates the Chinese fir wood flour-reinforced HDPE composite. The subscript *c* represents the Masson pine and Chinese fir wood flour-reinforced HDPE composite, which is comprised of various proportions of the Masson pine and Chinese fir wood flour-reinforced HDPE composite. *α* and *β* represent flour orientation factors; *α* is 1, *β* is 0.4 [27,28,44,45].

Based on data and curve trend analysis, a predictive model describing the mechanical properties of the Masson pine and Chinese fir wood flour-reinforced HDPE composite was established. The formula is as follows:

(9) $$P_{c}=\lambda P_{1}V_{1}+\left(2-\lambda \right)P_{2}V_{2}$$

In the above formula, *P* represents mechanical properties, and *λ* signifies the contribution factor of individual components to these mechanical properties. Specifically, in this study, it represents the role of the Masson pine wood flour-reinforced HDPE composite in the mechanical properties of the combined Masson pine and Chinese fir wood flour-reinforced HDPE composites. The term (2 − *λ*) represents the contribution of the Chinese fir wood flour-reinforced HDPE composite in the mechanical properties of composites reinforced with both Masson pine and Chinese fir wood flour.

(2) Comparison of Simulation Effects of Mechanical Models

The predicted values of the ROM model, IROM model, Hirsch model, and improved model were each compared with the test values, and the sum squared error (SSE) was calculated and compared.

SSE is calculated as follows:

(10) $$SSE={\sum_{i=1}^{i=7}\left(P_{\mathrm{model}}^{i}-P_{\mathrm{experiment}}^{i}\right)}^{2}$$

In the above formula, *P_model_* represents the simulated value of mechanical properties, while *P_experiment_* indicates the actual mechanical property values measured experimentally. *SSE* reflects the simulation effect of the model, and the smaller the *SSE*, the closer the predicted values are to the test value, indicating a more effective simulation performance of the model.

#### 2.3.8. Simulation and Comparison of Creep Performance

(1) Simulation of Creep Performance

The two-parameter index model, Findley index model, and modified ExpAssoc function were employed to predict the creep performance of the HDPE composite reinforced with Masson pine and Chinese fir wood flour. A comparison of the parameter standard deviation and simulation effectiveness was performed across the three models.

The formula for the Findley index model is as follows:

(11) $$\varepsilon \left(t\right)=a+bt^{c}$$

The formula for the two-parameter index model is as follows:

(12) $$\varepsilon \left(t\right)=bt^{c}$$

In the above formula, $\varepsilon \left(t\right)$ (mm/mm) represents the strain of the HDPE composite reinforced with Masson pine and Chinese fir wood flour at time *t* (h), *a* represents the initial elastic strain (mm/mm), *b* denotes the instantaneous creep strain (mm/mm), and *c* represents the time index. *t* represents the time (h).

The revised ExpAssoc model formula is as follows:

(13) $$\varepsilon \left(t\right)=a+d_{1}*\left[1-\exp\left(-d_{3}t\right)\right]+d_{2}*\left[1-\exp\left(-d_{4}t\right)\right]$$

In the above formula, *d_1_* and *d_2_* are strain coefficients, while *d_3_* and *d_4_* are time coefficients.

(2) Comparison of Creep Model Simulation Effects

The parameters, standard deviations, and simulation effects of the two-parameter index model, Findley index model, and the revised ExpAssoc function model were compared, and the creep variance analysis and other calibrations were carried out.

## 3. Results and Discussion

### 3.1. Effect of Mass Ratio of Mixed Wood Flour on Physical Properties of WPCs

#### 3.1.1. Effect of Mass Ratio of Mixed Wood Flour on Density of WPCs

The density values of the seven HDPE composite samples, reinforced with Masson pine wood flour and Chinese fir wood flour, are presented in Table 2. The density of the composites ranged from 1.165 to 1.197 g/cm^3^, with a variation of less than 3%. This indicates that replacing Chinese fir wood flour with Masson pine wood flour had scanty effect on the overall density of the composites.

#### 3.1.2. Effect of Mass Ratio of Mixed Wood Flour on Surface Color of WPCs

The results of the surface color tests for the seven composites are shown in Table 2. Among them, the Masson pine wood flour-reinforced HDPE composite had the highest surface brightness value. As the mass ratio of Masson pine to Chinese fir wood flour decreased, the surface brightness value of the composites gradually decreased. The surface brightness value and yellow–blue axis chromaticity index of the Chinese fir wood flour-reinforced HDPE composite were the smallest, and were only 70.44% and 64.36% of those in the Masson pine wood flour-reinforced HDPE composite. With an increase in the ratio of Masson pine wood flour to Chinese fir, both the red–green and yellow–blue axis chromaticity index of the composites increased. When five-sixths of the Chinese fir wood flour was replaced by Masson pine wood flour (WPC2), the composite had the maximum values for the red–green and yellow–blue axis chromaticity index, measuring 8.90 and 19.34, respectively. By changing the mass ratio of Masson pine to Chinese fir wood flour, composites with different colors can be prepared to meet the requirements of different places and different consumer groups for the appearance of decoration materials. At the same time, the surface color test of the Masson pine wood flour and Chinese fir wood flour-reinforced polymer composites will provide a foundation for future studies on their aging performance.

#### 3.1.3. Effect of Mass Ratio of Mixed Wood Flour on Dimensional Stability of WPCs

The test results for the dimensional stability of seven composites are shown in Table 2. For WPCs, the water absorption rate is an important performance index, as high water absorption can lead to corrosion and mold growth, which negatively impacts their physical and mechanical properties, ultimately reducing their durability and safety [46].

The 24 h water absorption rate of the seven WPCs ranged from 2.1% to 2.7%, indicating that altering the mass ratio of Masson pine to Chinese fir wood flour has minimal impact on the water absorption of the composites. However, notable differences were observed in the thickness swelling of the composites when exposed to water. The 24 h water absorption thickness swelling rate of the Chinese fir wood flour-reinforced HDPE composite was only 1.411%. When one-sixth of the Chinese fir wood flour was replaced with Masson pine wood flour (WPC6), the thickness swelling rate increased by 142.86%, and it continued to rise as more Chinese fir wood flour was substituted with Masson pine wood flour. The composite reinforced solely with Masson pine wood flour (WPC1) showed the highest thickness swelling rate, which was 3.43 times that of the Chinese fir wood flour-reinforced HDPE composite. A study by Zhao et al. [15] also showed that the water absorption rate and thickness swelling rate of WPCs made from Masson pine fibers are higher than those made from Chinese fir fibers. The superior dimensional stability of the WPC reinforced with Chinese fir wood flour was primarily due to the lower surface polarity and hydrophilicity of Chinese fir wood compared to Masson pine, making the WPC reinforced with Chinese fir wood flour less likely to adsorb polar molecules such as water.

### 3.2. Effect of Mass Ratio of Mixed Wood Flour on Mechanical Properties of WPCs

**Figure 2:**
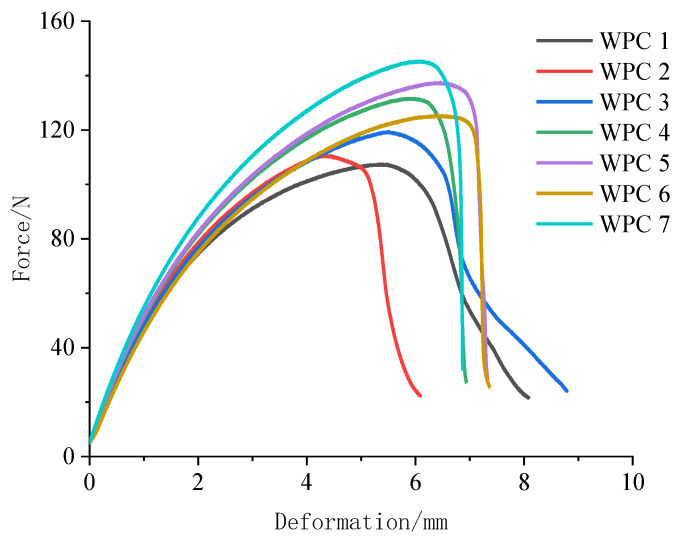
Force–deformation curves for WPCs under bending stress.

**Figure 3:**
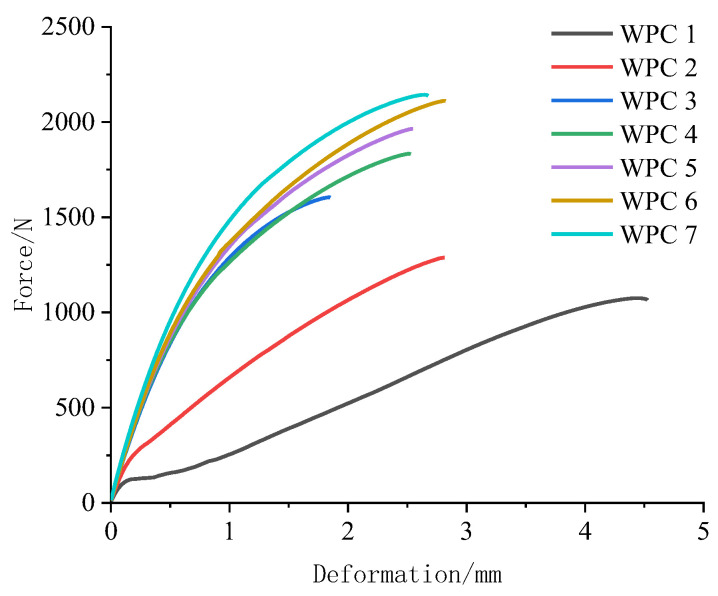
Force–deformation curves for WPCs under tension stress.

Table 3 presents the test results of the mechanical properties of seven kinds of *WPCs*. Figure 2 and Figure 3 show the force–deformation curves for WPCs under bending and tension stress. It was observed that the composite reinforced with Masson pine wood flour exhibited the worst mechanical properties. As the content of Chinese fir wood flour increased, the maximum load the composite could withstand before failure gradually improved under both bending and tensile forces, and the force-to-deformation ratio also progressively enhanced. Notably, the Chinese fir wood flour-reinforced HDPE composite exhibited the highest mechanical performance, with increases of 47.58%, 25.00%, 92.52%, 120.00%, and 70.03% in bending strength and modulus, tensile strength and modulus, and impact strength, respectively, compared to the composite reinforced with Masson pine wood flour. This improvement is attributed to the critical role of interface bonding between the wood and resin in WPCs. A stronger interface bond enables better cross-linking between the wood and matrix under load, allowing for more effective stress transfer and dispersion between the resin and wood components [47].

Among the seven composites, the Chinese fir wood flour-reinforced HDPE composite had the best physical and mechanical properties. This is mainly due to the negative impact of the resin on the interface bonding during the high-temperature melting process of the Masson pine wood, whereas the Chinese fir wood flour had a higher surface free energy (both total and non-polar free energy) compared to the Masson pine wood flour, resulting in a stronger bond with the HDPE matrix [46].

Although the mechanical properties of the seven composites are different, all of them met the requirements of GB/T 24137-2009 [48] for the bending strength and modulus of wood–plastic decorative panels (the average bending strength should be ≥20 MPa, the minimum value should be ≥16 MPa, and the bending modulus should be ≥1.8 GPa). Masson pine has a wider distribution and lower cost compared to Chinese fir. Therefore, in the production of wood–plastic decorative panels, Masson pine wood flour can be used to partially or fully replace Chinese fir wood flour. This substitution not only leverages the resource advantages of Masson pine wood but also reduces the consumption of Chinese fir wood, resulting in greater economic benefits.

### 3.3. Effect of Mass Ratio of Mixed Wood Flour on Dynamic Mechanical Properties of WPCs

#### 3.3.1. Effect of Mass Ratio of Mixed Wood Flour on Storage Modulus of WPCs

**Figure 4:**
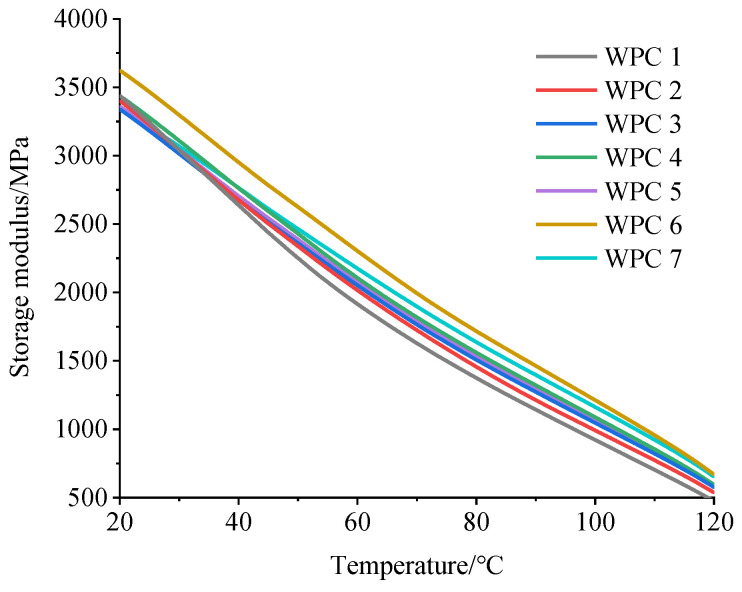
Storage modulus of WPCs as a function of temperature.

Figure 4 shows the storage modulus curves for seven kinds of HDPE composites reinforced with Masson pine wood flour and Chinese fir wood flour as the temperature increases.

The storage modulus of WPCs represents the ability of the composites to store elastic strain, and its value reflects the stiffness of WPCs. With the increase in temperature, the storage modulus of WPCs decreased because the higher temperature strengthened the activity capacity of the polymer molecules and reduced the elasticity of the composites. On the other hand, as the mass ratio of Masson pine wood flour to Chinese fir wood flour increased, the storage modulus of the composite showed a decreasing trend, which indicated that as the proportion of Masson pine wood flour increased, the interface compatibility between the wood flour and HDPE deteriorated, weakening the connection between polymer chains and making them easier to mobilize. Eventually, the elastic modulus and strength of the composites had deteriorated, consistent with the test results of the bending mechanical properties tests.

#### 3.3.2. Effect of Mass Ratio of Mixed Wood Flour on Loss Modulus of WPCs

**Figure 5:**
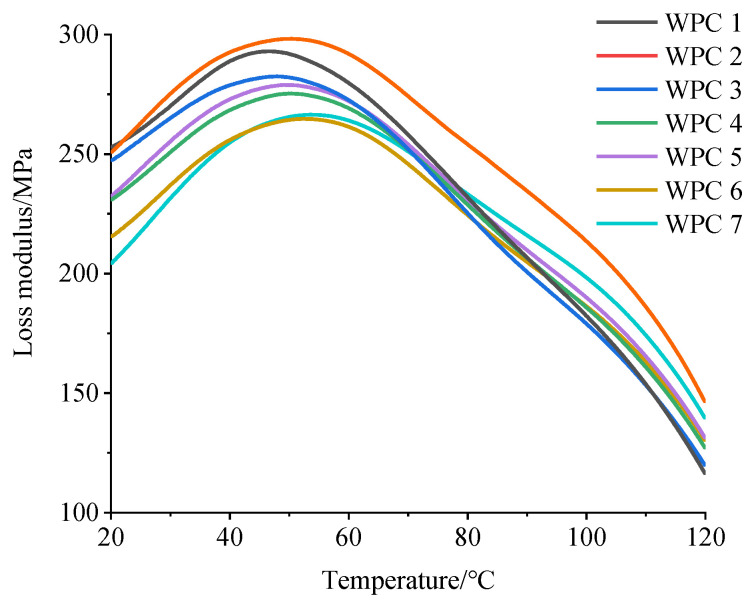
Loss modulus of WPCs as a function of temperature.

Figure 5 illustrates the loss modulus curves for HDPE composites reinforced with Masson pine wood flour and Chinese fir wood flour as the temperature rises.

The loss modulus of the WPCs increased with a higher content of Masson pine wood flour in the wood flour mixture. When the temperature reached approximately 50 °C, relaxation transition peaks appeared successively in the WPCs, with the highest loss modulus observed in the HDPE composite reinforced with Masson pine wood flour. Moreover, a smaller mass ratio of Masson pine wood flour to Chinese fir wood flour resulted in lower energy requirements for the composites to melt and flow.

#### 3.3.3. Effect of Mass Ratio of Mixed Wood Flour on Loss Tangent of WPCs

**Figure 6:**
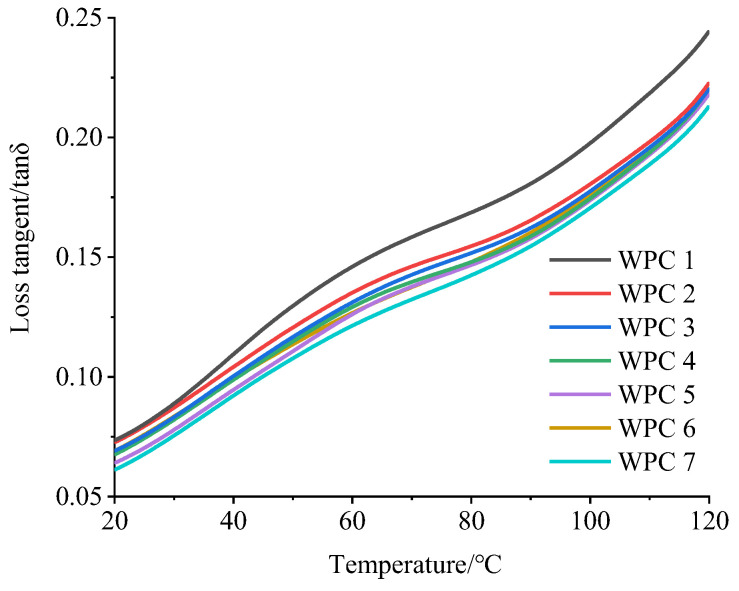
Loss tangent of WPCs as a function of temperature.

Figure 6 shows the loss tangent curves of the seven kinds of WPCs as the temperature increases.

The loss tangent, defined as the ratio of the loss modulus to the storage modulus, reflects the flexibility of the materials. The loss tangent value of the WPCs increased gradually with increasing temperature. This is due to the increased amplitude of molecular chain movement in HDPE as the temperature rises, leading to greater friction loss between them. Between 20 °C and 120 °C, the loss tangent values of the seven kinds of WPCs remained below 1, with no peak observed, indicating that the glass transition temperature of the WPCs had not been reached, and they remained in a hard, glass state. As the mass ratio of Masson pine wood flour to Chinese fir wood flour decreased, the loss tangent value gradually decreased, indicating a reduction in toughness and a more pronounced elastic behavior. This was mainly due to the decreased mass ratio of Masson pine wood flour to Chinese fir wood flour, which enhanced the bond strength between the wood flour and the plastic matrix. Under dynamic loading, the wood flour absorbed more energy, resulting in reduced energy loss, which further reflected the better interface bonding performance between the Chinese fir wood flour and HDPE. Under the condition of 25 °C, as the mass ratio of Masson pine wood flour to Chinese fir wood flour increased, the WPCs became more brittle, leading to a decrease in impact resistance, consistent with the impact test results.

### 3.4. Effect of Mass Ratio of Mixed Wood Flour on Creep Properties of WPCs

#### 3.4.1. The Bending Limit Load and 10% Stress Level of WPCs

The bending ultimate loads of the seven kinds of WPCs reinforced with Masson pine wood flour and Chinese fir wood flour were obtained through a three-point bending test, as listed in Table 4. The creep load *P* was set at 10% of the ultimate load, and the corresponding stress was calculated according to Formula (7), with the results also listed in Table 4. As the proportion of Chinese fir wood flour in the hybrid wood flour mixture increased, the ultimate load gradually increased, which is consistent with the results of the bending properties tests.

#### 3.4.2. Effect of Mass Ratio of Mixed Wood Flour on Creep Behaviour of WPCs

**Figure 7:**
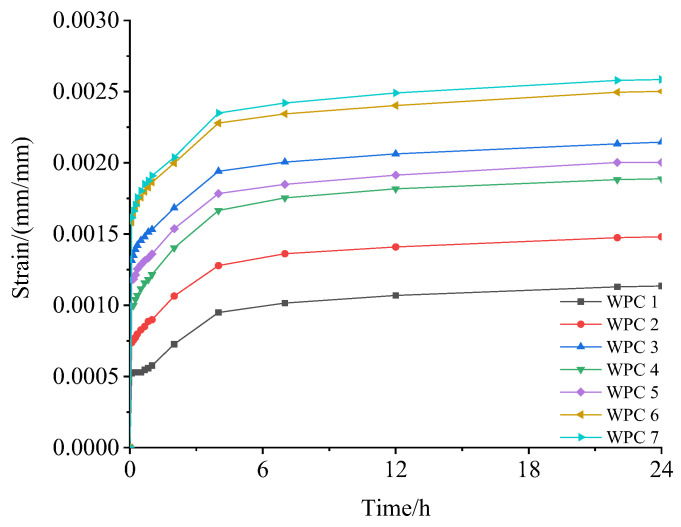
Strain–time curves of WPCs at 24 h.

At the 10% stress level, the changes in strain of the seven different WPCs over time are shown in Figure 7. As can be seen from Figure 7, the creep process of WPCs can be divided into two stages, namely the initial stage and the second stage. In the initial stage, the WPCs exhibit elastic strain and recoverable strain instantaneously, with a decreasing creep rate, which reflects the stability of the materials. The second stage was the transition stage, where the strain rate slowed and reached equilibrium, resulting in time-dependent reversible strain and residual strain. During this stage, the creep rate tended to be constant, and the strain exhibited a linear relationship with time.

Among the seven kinds of WPCs at the 10% stress level, the worst creep performance was observed in the Chinese fir wood flour-reinforced HDPE composite (WPC 7), whose 24 h strain ε_(24 h)_ was 0.002 585 mm/mm (Table 5). With the increase in the mass ratio of Masson pine wood flour and Chinese fir wood flour, the strain of the composites decreased on the whole, with the reduction becoming more pronounced. For instance, when the Chinese fir wood flour content in the mixed wood flour reached 83.33% (WPC 6), the ε_(24 h)_ was only 3.21% lower than that of the Chinese fir wood flour-reinforced HDPE composite (WPC 7). However, as the proportion of Chinese fir wood flour continued to decrease (66.67% for WPC 5, 50.00% for WPC 4, and 33.33% for WPC 3), the ε_(24 h)_ of the composites decreased significantly, measuring 0.002 002 mm/mm for WPC5, 0.001 888 mm/mm for WPC4, and 0.002 145 mm/mm for WPC3. When the proportion of Chinese fir wood flour in the mixed wood flour dropped below 16.67%, the ε_(24 h)_ of composites decreased significantly once again. In this case, the ε_(24 h)_ for WPC 2 was only 57.29% that of WPC 7 (the Chinese fir wood flour-reinforced HDPE composite). The best creep performance was observed in the Masson pine wood flour-reinforced HDPE composite (WPC 1), whose ε_(24 h)_ was only 0.001 135 mm/mm, representing 43.91% of the strain observed in WPC 7.

While the addition of Chinese fir wood improved the mechanical properties of the WPCs, it negatively impacted their creep characteristics. This is attributed to the relatively low 10% stress level, which was insufficient to break the interface bond between wood and polymer [49,50,51]. On the other hand, WPCs reinforced with Masson pine wood flour had a better ability to sustain small continuous loads. Considering that Masson pine wood flour is less expensive than Chinese fir wood flour, it can be used as a cost-effective substitute in applications involving low loads. This substitution enhances creep resistance while reducing material costs.

### 3.5. Simulation and Comparison of Mechanical Properties and of Creep Properties of WPCs

#### 3.5.1. Simulation and Comparison of Mechanical Properties of WPCs

(1) Simulation of Mechanical Properties of WPCs

The test results of the mechanical properties of WPCs reinforced with Masson pine wood flour and Chinese fir wood flour were substituted into the ROM model, the IROM model, the Hirsch model, and the improved model. The simulated values for bending strength (Table 6), bending modulus (Table 7), tensile strength (Table 8), tensile modulus (Table 9), and impact strength (Table 10) of hybrid wood flour composites were obtained.

The contribution factor (*λ*) of the model was fitted based on the mechanical properties of WPCs and the associated Formula (12). When *λ* is less than 1, it indicates that Chinese fir wood flour contributes more significantly to the mechanical properties of the composites, while when *λ* equals 1, it suggests that both wood types contribute equally. When *λ* exceeds 1, it signifies that Masson pine wood flour exerts a greater influence. For bending strength and modulus, the λ values were 0.977 and 1.034, respectively, while for tensile strength and modulus, they were 0.940 and 0.930, respectively. For impact strength, λ was 0.942. For the bending strength, tensile strength, tensile modulus, and impact strength of WPCs, λ was less than 1, indicating that the influence of Chinese fir wood flour on these properties of WPCs was greater than that of Masson pine wood flour. Among these, Chinese fir wood flour had the largest effect on tensile properties, followed by impact strength, with the least effect on bending strength. However, for bending modulus, λ was greater than 1, which indicates that the effect of Masson pine wood flour was more pronounced, approximately equivalent to the influence of Chinese fir wood flour on bending strength.

On the other hand, it can be seen from the prediction model that the type of wood flour had the greatest influence on the tensile properties of the composites, followed by impact strength, with bending properties having the least influence.

(2) Comparison of Mechanical Models of WPCs

The SSE of various mechanical properties in the ROM model, IROM model, Hirsch model, and the improved model were calculated by using the SSE formula, and are listed in Table 11. Among the three traditional models, the ROM model performed the best, while the IROM model had the lowest accuracy, with the Hirsch model ranking in the middle for simulating the bending strength, tensile strength, tensile modulus, and impact strength of WPCs. However, for the bending modulus, the simulation results of the three traditional models were similar, with the IROM model performing the best, followed by the Hirsch model, while the ROM model performed the worst.

By comparing the SSE values of the improved model and the three traditional models, it was found that the improved model showed superior performance in bending strength, tensile strength, tensile modulus, and impact strength. Analysis of variance showed that the improved model has more significant advantages in predicting tensile strength, demonstrating its capability to accurately estimate the mechanical properties (especially the tensile properties) of mixed wood flour-reinforced HDPE composites (WPC2-6) by using the known mechanical properties of two single wood flour-reinforced HDPE composites (WPC1 and WPC7).

#### 3.5.2. Simulation and Comparison of Creep Properties of WPCs

(1) Simulation of Creep Properties of WPCs

Based on the 24 h creep data for WPCs reinforced with Masson pine and Chinese fir wood flour under a stress level of 10% of their respective maximum load, the parameters of the Findley index model and the two-parameter index model were fitted, with the results and simulation effects displayed in Table 12 and Table 13.

The modified model, based on the ExpAssoc function, was used to simulate the 24 h creep data of WPCs at 10% stress level. The fitting results and simulation effects are shown in Table 14.

(2) Comparison of Creep Models of WPCs

The creep behavior of WPCs reinforced with Masson pine and Chinese fir wood flour was simulated and compared using the two-parameter index model and the Findley index model. Variance analysis of the creep data showed that both models effectively simulated the creep behavior of the composites. However, the Findley index model had a better simulation effect than the two-parameter index model, with *R*^2^ values all above 0.93, and the highest value reaching 0.972 3.

A further comparison was conducted between the Findley index model and the modified ExpAssoc function model. The modified ExpAssoc function model exhibited better fitting for the 24 h creep curve of the composites than the Findley index model, with all *R*^2^ values exceeding 0.99 and the highest value reaching 0.998 8.

**Figure 8:**
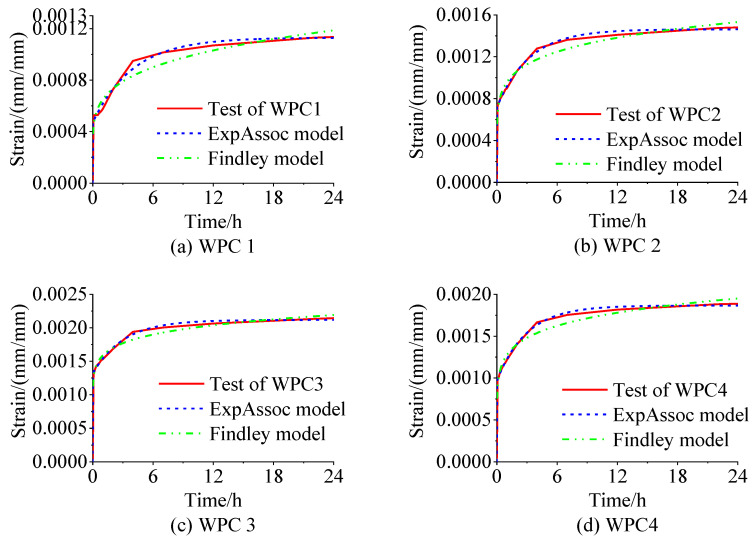

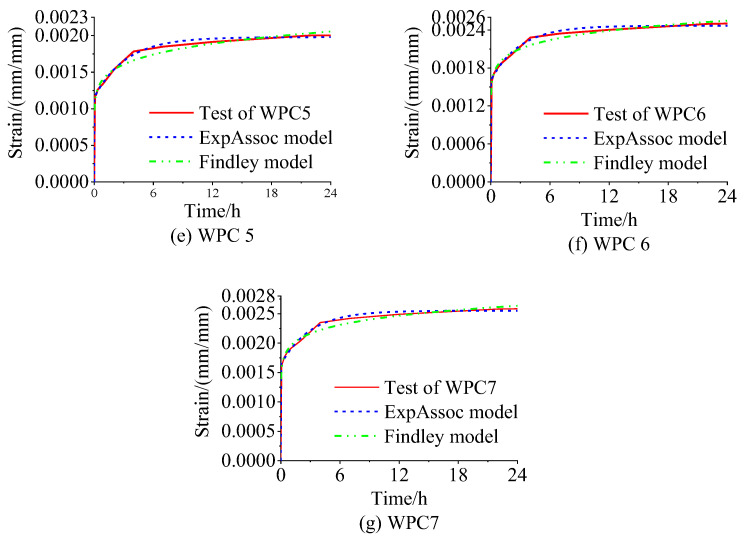
Creep testing and simulation curves of WPC samples.

Furthermore, a comparison of the experimental creep test curves with the fitted curves from the Findley index model, and the modified ExpAssoc function model (as shown in Figure 8) showed that the results from the modified ExpAssoc function model aligned more closely with the experimental test curves. This consistency further validates the accuracy of the modified ExpAssoc function model.

Utilizing mathematical models to fit the mechanical properties and creep behavior of WPCs can be very helpful in predicting their performance, which helps to understand the influence of component proportions on the performance of the final product, and allows for the optimization of mechanical properties and creep resistance of WPCs by adjusting the formulation or processing conditions. During the development and production process, mathematical models can partially replace a large number of physical experiments, thereby saving considerable time and costs. Furthermore, in the design phase, these models can assess whether the material meets application requirements under specific load conditions, ensuring that the manufactured product is more stable and reliable.

## 4. Conclusions

This study investigated the physical, mechanical, and creep properties of WPCs reinforced with Masson pine and Chinese fir wood flour. Several models, including the ROM model, the IROM model, the Hirsch model, and the improved model, were used to simulate the mechanical properties of the WPCs. The creep properties were modeled using the Findley index model, the two-parameter index model, and the modified ExpAssoc model. Simulation results from these models were compared with experimental data. The main results are as follows.

The ratio of Masson pine to Chinese fir wood flour had little effect on the density of WPCs, with variations not exceeding 3%. The surface brightness value, red–green and yellow–blue axis chromaticity index, and the 24 h water absorption thickness swelling rate of the composites all decreased as the mass ratio of Masson pine to Chinese fir decreased. Increasing the proportion of Chinese fir wood flour in the mixture improved the mechanical properties of the composites. Specifically, WPCs reinforced with Chinese fir wood flour had the highest bending strength and modulus, tensile strength and modulus, and impact strength, with improvements of 47.58%, 25.00%, 92.52%, 120.00%, and 70.03%, respectively, compared to WPCs reinforced with Masson pine wood flour. As the mass ratio of Masson pine wood flour increased, the storage modulus of the composites showed a decreasing trend, while the loss modulus increased. Among the seven types of WPCs, the composite reinforced with Chinese fir wood flour showed the worst creep performance at a 10% stress level, with a 24 h strain ε_(24 h)_ of 0.002 585 mm/mm. With the increase in the mass ratio of Masson pine wood flour and Chinese fir wood flour, the strain of the composites decreased on the whole, and the decreasing range increased gradually. Compared with the ROM, IROM, and Hirsch models, the modified model yielded the best simulation results. In terms of creep behavior, the modified ExpAssoc model proved superior to both the Findley index model and the two-parameter index model.

## Figures and Tables

**Table 1:** Composition mass ratio of wood flour-reinforced polymer composite.

No.	Masson Pine Wood Flour/%	Chinese Fir Wood Flour/%	HDPE/%	MAPE and Wax/%
WPC1	60	0	36	4
WPC2	50	10	36	4
WPC3	40	20	36	4
WPC4	30	30	36	4
WPC5	20	40	36	4
WPC6	10	50	36	4
WPC7	0	60	36	4

**Table 2:** Physical properties of hybrid wood flour-reinforced HDPE composites.

No.	Density/g·cm^−3^	Surface Lightness (L*)	Red–Green Axis Chromatic (a*)	Yellow–Blue Axis Chromatic Index (b*)	Total Color Change (∆E*)	24 h Water Absorption Rate/%	24 h Water Absorption Thickness Swelling Rate/%
WPC1	1.181 (0.008)	56.74 (0.821)	5.77 (0.280)	16.44 (0.333)	—	2.148 (0.518)	4.836 (0.199)
WPC2	1.197 (0.013)	47.86 (0.843)	8.90 (0.418)	19.34 (0.665)	9.85	2.361 (0.314)	3.802 (1.120)
WPC3	1.194 (0.008)	45.60 (0.651)	8.50 (0.169)	15.86 (0.844)	11.48	2.710 (0.283)	3.699 (1.414)
WPC4	1.188 (0.006)	43.41 (0.735)	8.47 (0.219)	14.40 (0.228)	13.75	2.689 (0.254)	3.621 (1.147)
WPC5	1.182 (0.004)	43.20 (0.254)	8.03 (0.311)	12.90 (0.639)	14.18	2.521 (0.430)	3.499 (1.178)
WPC6	1.189 (0.017)	40.66 (0.389)	7.78 (0.252)	11.72 (0.480)	16.88	2.432 (0.266)	3.419 (2.319)
WPC7	1.165 (0.099)	39.97 (0.212)	7.28 (0.254)	10.58 (0.461)	17.83	2.412 (0.380)	1.411 (0.576)

**Table 3:** Mechanical properties of hybrid wood flour-reinforced HDPE composites.

No.	Bending Strength/MPa	Bending Modulus/GPa	Tensile Strength/MPa	Tensile Modulus/GPa	Impact Strength/kJ·m^−2^
WPC1	43.3 (0.777)	3.2 (0.007)	21.4 (0.971)	1.0 (0.491)	6.94 (0.363)
WPC2	47.0 (0.936)	3.3 (0.078)	25.3 (0.416)	1.2 (0.591)	8.36 (0.225)
WPC3	50.3 (0.944)	3.4 (0.028)	29.5 (0.480)	1.9 (0.055)	9.11 (0.369)
WPC4	56.0 (0.316)	3.4 (0.198)	33.9 (0.129)	1.9 (0.025)	10.34 (0.303)
WPC5	57.6 (0.871)	3.5 (0.049)	36.2 (0.431)	1.9 (0.035)	10.73 (0.106)
WPC6	61.6 (0.449)	3.8 (0.062)	39.3 (0.805)	2.0 (0.283)	11.53 (0.926)
WPC7	63.9 (1.158)	4.0 (0.023)	41.2 (0.872)	2.2 (0.049)	11.80 (0.582)

**Table 4:** Bending ultimate load, creep load, and stress of hybrid wood flour-reinforced HDPE composites.

No.	Bending Ultimate Load/N	Creep Load/N	Stress/MPa
WPC1	317.355 (2.554)	31.60	4.446
WPC2	352.559 (1.312)	34.30	4.941
WPC3	372.165 (2.279)	36.26	5.372
WPC4	407.201 (6.035)	40.18	6.048
WPC5	424.302 (2.275)	41.16	6.202
WPC6	444.302 (1.026)	43.12	6.478
WPC7	467.540 (0.346)	46.06	6.893

**Table 5:** Strain of hybrid wood flour-reinforced HDPE composites at 1, 2, 4, 12 h, and 24 h.

No.	ε(1 h)	ε(2 h)	ε(4 h)	ε(12 h)	ε(24 h)
WPC1	0.000 577	0.000 727	0.000 949	0.001 069	0.001 135
WPC2	0.000 898	0.001 065	0.001 279	0.001 410	0.001 481
WPC3	0.001 531	0.001 684	0.001 940	0.002 063	0.002 145
WPC4	0.001 216	0.001 403	0.001 666	0.001 818	0.001 888
WPC5	0.001 360	0.001 537	0.001 784	0.001 914	0.002 002
WPC6	0.001 863	0.001 998	0.002 279	0.002 402	0.002 502
WPC7	0.001 909	0.002 038	0.002 350	0.002 491	0.002 585

**Table 6:** Experimental and simulated values of bending strength of WPCs.

No.	Experimental Bending Strength Values/MPa	Simulated Values/MPa			
		ROM Model	IROM Model	Hirsch Model	Improved Model
WPC1	43. 3 (0.777)	-	-	-	-
WPC2	47. 0 (0.936)	46. 73	45. 76	46. 15	46. 15
WPC3	50. 3 (0.944)	50. 17	48. 51	49. 17	49. 99
WPC4	56. 0 (0.316)	53. 60	51. 62	52. 41	53. 84
WPC5	57. 6 (0.871)	57. 03	55. 15	55. 91	57. 68
WPC6	61. 6 (0.449)	60. 47	59. 21	59. 71	61. 53
WPC7	63. 9 (1.158)	-	-	-	-

**Table 7:** Experimental and simulated values of bending modulus of WPCs.

No.	Experimental Bending Modulus Values/GPa	Simulated Values/GPa			
		ROM Model	IROM Model	Hirsch Model	Improved Model
WPC1	3. 2 (0.007)	-	-	-	-
WPC2	3. 3 (0.078)	3. 33	3. 31	3. 32	3. 40
WPC3	3. 4 (0.028)	3. 47	3. 43	3. 44	3. 49
WPC4	3. 4 (0.198)	3. 60	3. 56	3. 57	3. 59
WPC5	3. 5 (0.049)	3. 73	3. 69	3. 71	3. 68
WPC6	3. 8 (0.062)	3. 87	3. 84	3. 85	3. 77
WPC7	4. 0 (0.023)	-	-	-	-

**Table 8:** Experimental and simulated values of tensile strength of WPCs.

No.	Experimental Tensile Strength Values/MPa	Simulated Values/MPa			
		ROM Model	IROM Model	Hirsch Model	Improved Model
WPC1	21. 4 (0.971)	-	-	-	-
WPC2	25. 3 (0.416)	24. 70	23. 26	23. 84	24. 04
WPC3	29. 5 (0.480)	28. 00	25. 48	26. 49	27. 97
WPC4	33. 9 (0.129)	31. 30	28. 17	29. 42	31. 89
WPC5	36. 2 (0.431)	34. 60	31. 49	32. 73	35. 82
WPC6	39. 3 (0.805)	37. 90	35. 70	36. 58	39. 75
WPC7	41. 2 (0.872)	-	-	-	-

**Table 9:** Experimental and simulated values of tensile modulus of WPCs.

No.	Experimental Tensile Modulus Values/GPa	Simulated Values/GPa			
		ROM Model	IROM Model	Hirsch Model	Improved Model
WPC1	1.0 (0.491)	-	-	-	-
WPC2	1.2 (0.591)	1.20	1.10	1.14	1.17
WPC3	1.9 (0.055)	1.40	1.22	1.29	1.40
WPC4	1.9 (0.025)	1.60	1.38	1.47	1.64
WPC5	1.9 (0.035)	1.80	1.57	1.66	1.88
WPC6	2.0 (0.283)	2.00	1.83	1.90	2.12
WPC7	2.2 (0.049)	-	-	-	-

**Table 10:** Experimental and simulated values of impact strength of WPCs.

No.	Experimental Impact Strength Values/kJ·m^−2^	Simulated Values/kJ·m^−2^			
		ROM Model	IROM Model	Hirsch Model	Improved Model
WPC1	6. 94 (0.363)	-	-	-	-
WPC2	8. 36 (0.225)	7. 750	7. 452	7. 571	7. 529
WPC3	9. 11 (0.369)	8. 560	8. 044	8. 251	8. 520
WPC4	10. 34 (0.303)	9. 370	8. 740	8. 992	9. 511
WPC5	10. 73 (0.106)	10. 180	9. 567	9. 812	10. 502
WPC6	11. 53 (0.926)	10. 990	10. 567	10. 736	11. 493
WPC7	11. 80 (0.582)	-	-	-	-

**Table 11:** SSE values of ROM, IROM, Hirsch, and improved models.

Mechanical Properties	ROM Model	IROM Model	Hirsch Model	Improved Model
Bending strength	7.454 444	35.630 65	21.305 99	5.510 903
Bending modulus	0.104 444	0.0637 03	0.078 476	0.086 656
Tensile strength	13.890 00	88.329 28	50.693 65	8.296 94
Tensile modulus	0.350 000	0.880 745	0.627 106	0.327 024
Impact strength	2.209 600	6.802 397	4.651 539	1.780 155

**Table 12:** Two-parameter index model parameters and simulation results.

No.	Value of *a* (×10^−3^)	Standard Error of *a* (×10^−5^)	Value of *b* (×10^−2^ )	Standard Error of *b* (×10^−2^)	Residual Value of χ^2^ (×10^−8^)	Adj. *R*^2^
WPC1	0.72	5.42	12.2	3.44	3.859 7	0.385 6
WPC2	1.03	5.66	9.44	2.51	4.439 7	0.444 0
WPC3	1.64	3.83	7.51	1.05	2.961 3	0.787 9
WPC4	1.32	4.19	11.0	1.46	2.369 2	0.807 8
WPC5	1.49	4.89	7.86	1.48	3.397 5	0.655 0
WPC6	1.95	3.19	7.39	0.74	1.456 3	0.885 1
WPC7	1.99	2.93	7.98	0.67	1.215 3	0.918 8

**Table 13:** Findley index model parameters and simulation results.

No.	Value of *a* (×10^−4^)	Standard Error of *a* (×10^−4^)	Value of *b* (×10^−4^)	Standard Error of *b* (×10^−4^)	Value of *c* (×10^−3^)	Standard Error of *c* (×10^−2^)	Residual Value of χ^2^ (×10^−8^)	Adj. *R*^2^
WPC1	2.69	1.87	3.88	5.55	2.79	11.1	5.733 3	0.933 2
WPC2	2.52	3.37	1.48	1.71	1.90	8.03	4.696 2	0.957 7
WPC3	6.94	4.27	2.31	2.48	1.65	7.12	4.473 7	0.964 9
WPC4	2.81	5.19	2.54	3.00	1.66	7.85	6.802 8	0.957 8
WPC5	6.47	3.52	1.61	1.78	1.92	7.71	5.284 1	0.961 0
WPC6	7.98	5.09	3.31	3.25	1.47	6.21	4.135 0	0.972 3
WPC7	7.85	5.47	3.47	3.47	1.48	6.35	4.860 9	0.971 1

**Table 14:** Modified ExpAssoc function model parameters and simulation results.

No.	Value of *a*	Standard Error of *a* (×10^−5^)	Value of *d*_1_ (×10^−4^ )	Standard Error of *d*_1_ (×10^−5^)	Value of *d*_2_ (×10^−4^)	Standard Error of *d*_2_ (×10^−5^)	Residual Value of χ^2^ (×10^−9^)	Adj. *R*^2^
WPC1	2.31 × 10^−13^	3.36	6.60	2.46	4.73	3.73	11.278	0.991 8
WPC2	1.11 × 10^−16^	1.75	7.19	-	7.47	894	3.045 0	0.998 6
WPC3	7.12 × 10^−7^	2.49	13.2	2.77	7.99	1.76	6.181 4	0.998 4
WPC4	1.47 × 10^−8^	2.02	8.99	1.43	9.70	2.28	4.062 1	0.998 8
WPC5	1.29 × 10^−22^	2.61	8.24	1.86	11.60	2.94	6.809 4	0.998 1
WPC6	2.82 × 10^−9^	3.33	16.1	-	8.63	3.14	11.118	0.997 9
WPC7	9.13 × 10^−6^	3.63	16.3	4.22	9.18	2.59	13.172	0.997 6

## Data Availability

Data are contained within the article.
